# Missense Mutations in *CRYAB* Are Liable for Recessive Congenital Cataracts

**DOI:** 10.1371/journal.pone.0137973

**Published:** 2015-09-24

**Authors:** Xiaodong Jiaox, Shahid Y. Khan, Bushra Irum, Arif O. Khan, Qiwei Wang, Firoz Kabir, Asma A. Khan, Tayyab Husnain, Javed Akram, Sheikh Riazuddin, J. Fielding Hejtmancik, S. Amer Riazuddin

**Affiliations:** 1 Ophthalmic Genetics and Visual Function Branch, National Eye Institute, National Institutes of Health, Bethesda, MD, 20892, United States of America; 2 The Wilmer Eye Institute, Johns Hopkins University School of Medicine, Baltimore, MD, 21287, United States of America; 3 National Centre of Excellence in Molecular Biology, University of the Punjab, Lahore, 53700, Pakistan; 4 King Khaled Eye Specialist Hospital, Riyadh, 12329, Saudi Arabia; 5 Allama Iqbal Medical College, University of Health Sciences, Lahore, 54550, Pakistan; 6 National Centre for Genetic Diseases, Shaheed Zulfiqar Ali Bhutto Medical University, Islamabad, Pakistan; Tsinghua University, CHINA

## Abstract

**Purpose:**

This study was initiated to identify causal mutations responsible for autosomal recessive congenital cataracts in consanguineous familial cases.

**Methods:**

Affected individuals underwent a detailed ophthalmological and clinical examination, and slit-lamp photographs were ascertained for affected individuals who have not yet been operated for the removal of the cataractous lens. Blood samples were obtained, and genomic DNA was extracted from white blood cells. A genome-wide scan was completed with short tandem repeat (STR) markers, and the logarithm of odds (LOD) scores were calculated. Protein coding exons of *CRYAB* were sequenced, bi-directionally. Evolutionary conservation was investigated by aligning *CRYAB* orthologues, and the expression of *Cryab* in embryonic and postnatal mice lens was investigated with TaqMan probe.

**Results:**

The clinical and ophthalmological examinations suggested that all affected individuals had nuclear cataracts. Genome-wide linkage analysis suggested a potential region on chromosome 11q23 harboring *CRYAB*. DNA sequencing identified a missense variation: c.34C>T (p.R12C) in *CRYAB* that segregated with the disease phenotype in the family. Subsequent interrogation of our entire cohort of familial cases identified a second familial case localized to chromosome 11q23 harboring a c.31C>T (p.R11C) mutation. *In silico* analyses suggested that the mutations identified in familial cases, p.R11C and p.R12C will not be tolerated by the three-dimensional structure of *CRYAB*. Real-time PCR analysis identified the expression of *Cryab* in mouse lens as early as embryonic day 15 (E15) that increased significantly until postnatal day 6 (P6) with steady level of expression thereafter.

**Conclusion:**

Here, we report two novel missense mutations, p.R11C and p.R12C, in *CRYAB* associated with autosomal recessive congenital nuclear cataracts.

## Introduction

Congenital cataracts are the principal cause of visual impairment in children as they responsible for one-third of cases of blindness in infants worldwide [1,2]. The ocular lens focus the light on the retina and the loss of transparency of the lens comprises this important function, which could lead to permanent blindness, especially during the early developmental periods. Nearly, one-third of the total cases congenital cataract are familial with both autosomal dominant and autosomal recessive inheritance [3]. Congenital cataracts are genetically heterogeneous with genetic loci for both autosomal dominant cataracts (adCC) and autosomal recessive cataracts (arCC) have been localized.

The last decade witnessed localization of multiple loci for congenital cataracts and taken together a total 16 arCC loci have so far been reported [4–19]. Of these genetic loci, causal mutations in eph-receptor type-A2 (*EPHA2)*, connexin50 (*GJA8)*, FYVE and coiled-coil domain containing 1 (*FYCO1*), glucosaminyl (N-acetyl) transferase 2 (*GCNT2)*, heat-shock transcription factor 4 (*HSF4*), lens intrinsic membrane protein 2 (*LIM2*), beaded filament structural protein 1 (*BFSP1*), crystallin alpha A (*CRYAA*), crystallin beta B1 (*CRYBB1*), and crystallin beta B3 (*CRYBB3*) have been identified [4,6,8,11,13–17,20].

Crystallins constitute nearly 95% of the soluble protein of the vertebrate eye lens as high concentrations of tightly packed crystalline proteins are required for lens transparency and its physiological function [21]. They are sub-divided into three classes, namely alpha, beta and gamma crystallins based upon their elution profile on gel exclusion chromatography. *CRYAB* is located on chromosome 11q23 and encodes for a member of the small heat-shock protein family comprising of 175 amino acid protein [22]. The CRYAB protein is expressed in multiple tissues including the ocular lens, heart, skeletal muscle, kidney, lung, and glia in the central nervous system [22].

Here, we report two consanguineous familial cases with multiple individuals in both families having congenital cataracts. Ophthalmic examination with a slit lamp confirmed nuclear cataracts present in the affected individuals. The genome-wide linkage or exclusion analysis localized the disease phenotype in two consanguineous familial cases to chromosome 11q23. Bi-directional sequencing identified missense mutations in *CRYAB* that segregated with the disease phenotype in their respective families and were absent in ethnically matched controls chromosomes. To the best of our knowledge, this is the first report identifying mutations in *CRYAB* associated with congenital cataracts in Pakistani families.

## Materials and Methods

### Recruitment and Clinical Assessment

A total of >200 consanguineous Pakistani families with non-syndromic cataract were recruited to identify new disease loci responsible for inherited visual diseases. The Institutional Review Board (IRB) of National Centre of Excellence in Molecular Biology, Lahore Pakistan, the CNS IRB of the National Eye Institute, Bethesda MD and Johns Hopkins University, Baltimore MD approved for this study. All participating family members provided informed written consent that has been endorsed by the respective IRBs and is consistent with the tenets of the Declaration of Helsinki. A detailed clinical and medical history was obtained from the individual families. The ophthalmic examination was performed with a slit-lamp and phtogrpahs were taken to record the ocular phenotype. A consent to publish ocular phenotype was obtained from the patient and/or the legal guardian. All participating members voluntarily provided blood sample of approximately 10 ml that was stored in 50 ml Sterilin® falcon tubes containing 400 μl of 0.5 M EDTA. Blood samples were stored at -20°C for long-term storage.

### Genomic DNA Extraction

The genomic DNAs were extracted from white blood cells using a non-organic modified procedure as described previously [23]. The concentration of the extracted genomic DNA was estimated using a SmartSpec plus Bio-Rad Spectrophotometer (Bio-Rad, Hercules, CA).

### Genome-Wide Scan and Exclusion Analysis

The Applied Biosystems MD-10 linkage mapping panels (Applied Biosystems, Foster City, CA) were used to complete a genome-wide scan for family PKCC001. Multiplex polymerase chain reaction (PCR) was completed as described previously [23]. PCR products were mixed with a loading cocktail containing HD-400 size standards (Applied Biosystems) and resolved in an Applied Biosystems 3100 DNA Analyzer. Genotypes were assigned using the Gene Mapper software from the Applied Biosystems. Exclusion analysis was completed for PKCC113 using closely spaced STR markers. The sequences of the primer pairs used for exclusion analysis and amplification conditions are available upon request

### Linkage Analysis

Linkage analysis was performed with alleles of PKCC001 obtained through the genome-wide scan and alleles of PKCC113 obtained through exclusion analysis using the FASTLINK version of MLINK from the LINKAGE Program Package [24,25]. Maximum LOD scores were calculated using ILINK from the LINKAGE Program Package. arCC was investigated as a completely penetrant disorder with an affected allele frequency of 0.001.

### Mutation Screening

The sequences of primer pairs used to amplify individual exons of *CRYAB* are available upon request. PCR reactions were completed in 10 μl volume containing 20 ng of genomic DNA. The reaction consisted of a denaturation step at 95°C for 5 min followed by a two-step touchdown procedure. The first step of 10 cycles consisted of denaturation at 95°C for 30 seconds, followed by a primer set specific annealing for 30 seconds (annealing temperature decrease by 1°C per cycle) and elongation at 72°C for 45 seconds. The second step of 30 cycles consisted of denaturation at 95°C for 30 seconds followed by annealing (annealing temperature -10°C) for 30 seconds and elongation at 72°C for 45 seconds, followed by a final elongation 72°C for 5 minutes.

The PCR primers for each exon were used for bi-directional sequencing using BigDye Terminator Ready reaction mix, according to the manufacturer’s instructions. The sequencing products were resolved on an ABI PRISM 3100 DNA analyzer (Applied Biosystems), and the results were analyzed using Applied Biosystems SeqScape software.

### Evolutionary Conservation

Evolutionary conservation of the amino acid Arg11 and Arg12 was investigated by aligning the protein sequence of CRYAB orthologues. The evolutionary conservation of amino acid and the possible effect of the amino acid substitution on the structure of the CRYAB protein was examined using SIFT (http://sift.jcvi.org ) and PolyPhen2 (http://genetics.bwh.harvard.edu/pph2/index.shtml) algorithms, respectively.

### Biophysical Characteristics

The polarity, optimized matching hydrophobicity, and hydropathicity of the wild type and mutant CRYAB proteins was examined using ProtScale, a bioinformatics tool on ExPASy Server (http://www.expasy.org/tools/protscale.html). Similarly, we used ProtScale to compute the isoelectric point (pI) and the molecular weight (Mw) of wild type and mutant CRYAB proteins. The machine-learning algorithm (mCSM) was employed to predict the deleterious effects of missense variants on CRYAB structure. The crystal structure of human CRYAB (PDB code 2YGD) was utilized to predict the impact on protein stability.

### Real-Time Expression Analysis

The use of mice in this study was approved by the Johns Hopkins Animal Care and Use committee (ACUC), and all protocols were performed in accordance with a protocol approved by the Johns Hopkins ACUC. Mouse lenses were obtained at different developmental stages including embryonic day 15 (E15), day 18 (E18), at birth, designated as (P0), postnatal day 3 (P3), day 6 (P6), day 9 (P9), day 12 (P12), day 14 (P14), day 21 (P21), day 28 (P28), day 42 (P42), day 56 (P56). Mice were first anesthetized with isoflurane and subsequently euthanized through cervical dislocation. The ocular tissue was extracted, and the lenses were isolated from the retina using forceps under a microscope. The lenses were divided into two pools, each representing a biological replicates for the respective developmental stage. Lenses were dissolved in TRIzol reagent (Invitrogen; Carlsbad, CA) immediately after isolation, and total RNA was extracted from each pool according to manufacturer’s instructions. The quality and quantity of the total RNA was determined on a NanoDrop Lite spectrophotometer (Thermo Scientific, Inc.). First-strand cDNA synthesis was completed using the Superscript III kit (Invitrogen) according to the manufacturer’s instructions. Quantitative real-time PCR analysis was performed on a STEP ONE ABI Real-Time PCR System using predesigned *Cryab* TaqMan expression assays (Applied Biosystems). *Gapdh* was used as an endogenous internal control. The 2^-ΔCT^ method was used to determine the relative expression normalized to *Gapdh* expression at each developmental stage.

## Results

**Fig 1:**
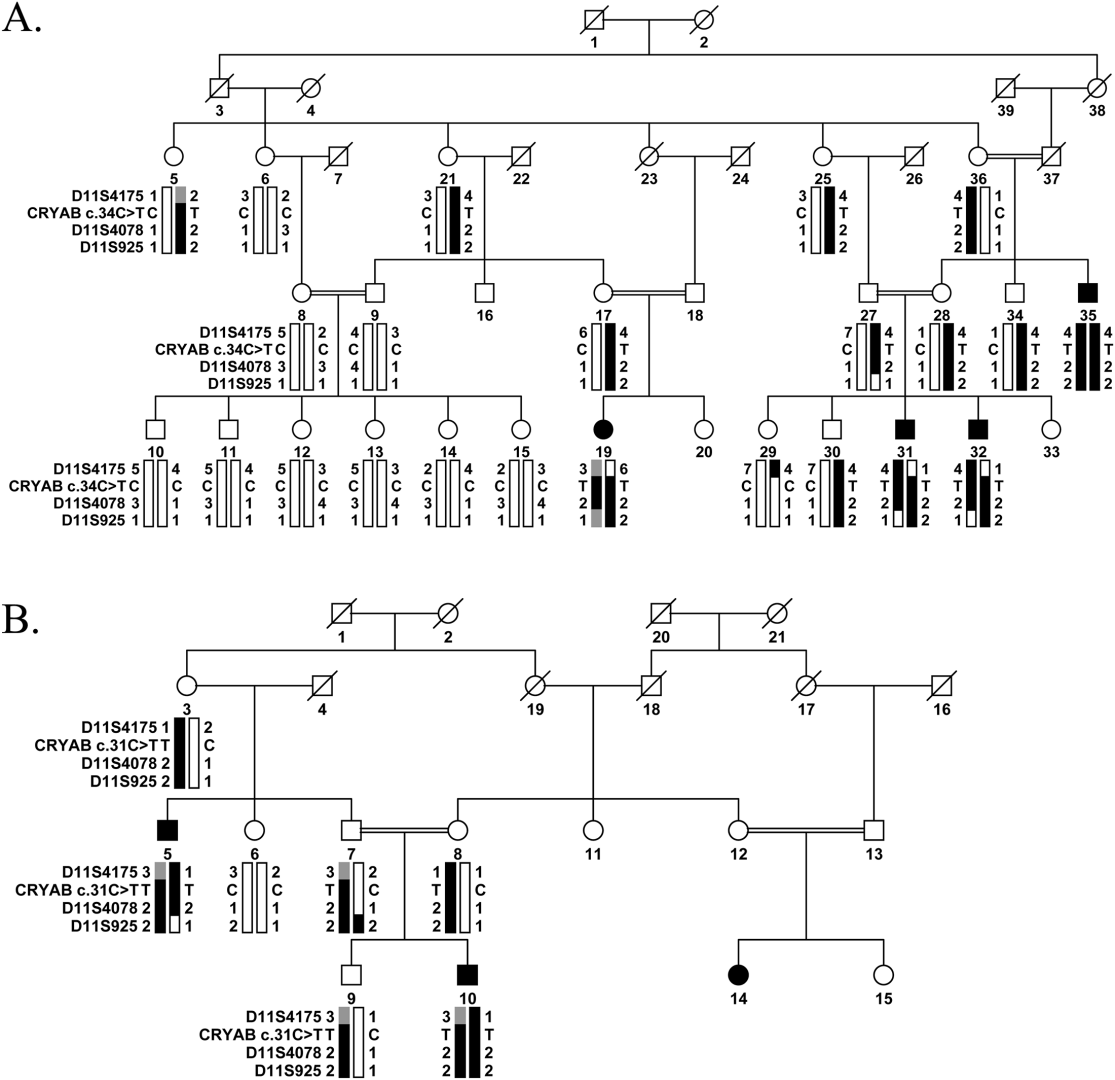
Pedigree drawing with haplotypes of chromosome 11q microsatellite markers. A) Family PKCC001 and B) family PKCC113 with alleles forming the risk haplotype are shown in black, heterozygous alleles part of the risk haplotype are shown in grey and alleles not cosegregating with cataract phenotype are shown in white. Squares: males; circles: females; filled symbols: affected individuals; the double line between individuals: consanguineous mating; and a diagonal line through a symbol: a deceased individual.

**Fig 2:**
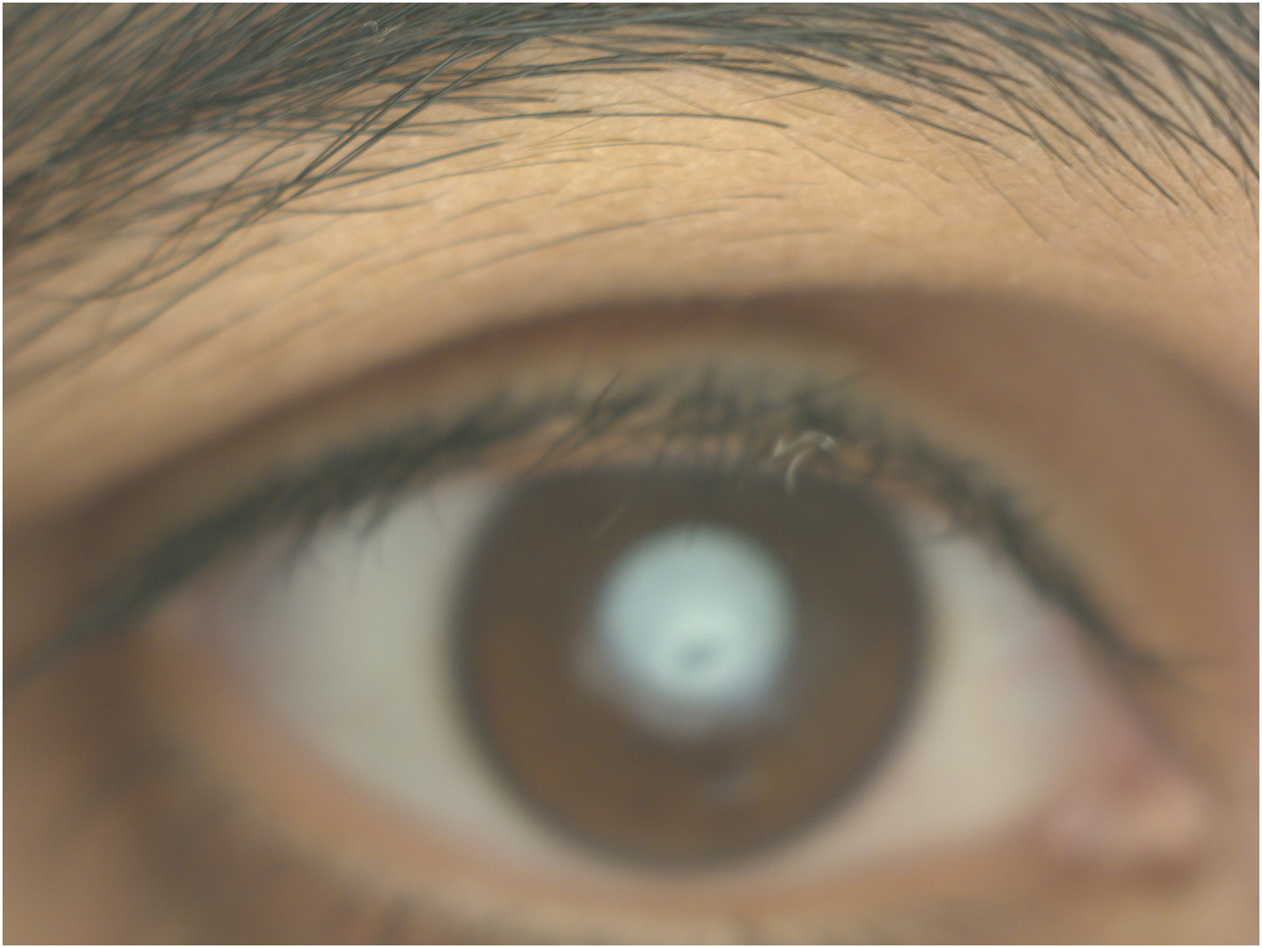
Slit lamp photographs of individual 19 of family PKCC001 illustrating nuclear cataracts.

An inbred large multigenerational family, PKCC001 with multiple individuals having congenital cataracts was recruited from the Punjab province of Pakistan to investigate the genetic basis of congenital cataracts (Fig 1A). A detailed medical history was obtained by interviewing all participating members, which ruled out any systemic abnormalities and/or extraocular phenotypes. According to the family elders, development of cataracts in affected individuals was first observed within a year after birth suggesting an early, perhaps a congenital onset. All affected individuals except individual 19 of family PKCC001 had been operated on to remove the cataractous lens prior to enrollment in the study and, therefore, we were unable to document their phenotype. An ophthalmic examination performed using a slit lamp microscopy revealed nuclear cataracts in individual 19 (Fig 2).

We were able to enroll a total of four affected individuals along with 19 unaffected members of PKCC001. The large numbers of enrollment augmented the power of the family to generate statistically significant two-point LOD scores during genome-wide linkage. Our theoretical estimates confirmed that PKCC001 can attain a maximum two-point LOD score of 5.30 at θ = 0. We completed a genome-wide scan and subsequently calculated two-point LOD scores. Surprisingly, we did not identify a single region of statistical significance (LOD >3) or suggestive linkage (LOD >2) across the entire genome except marker D19S414 on chromosome 19 yielding a two-point LOD score of 2.0 at θ = 0. However, markers adjacent to D19S414, D19S226 on the proximal and D19S220 on distal end produced highly negative two-point LOD scores ruling out the candidacy of chromosome 19q.

The lack of two-point LOD scores mimicking the theoretical potential of PKCC001 during the genome-wide scan, although surprising, but is certainly not new to us. We have witnessed large familial cases in our cohort yielding huge two-point LOD scores during theoretical simulations without presenting any peaks of significant and/or suggestive linkage during genome-wide scans. The most likely explanation being that a high degree of consanguinity and inbreeding for many generations may have reduced the critical disease interval below 10 cM resolution of the MD-10 panel.

Thus, we re-evaluated our linkage data and identified a region of chromosome 11q23 with two adjacent panel markers D11S4175 and D11S925 yielding positive two-point LOD scores (Table 1A). To establish linkage to chromosome 11q23 region, we chose a marker between D11S4175 and D11S925 from the MD-5 panel, D11S4078 that yielded a two-point LOD score of 4.29 at θ = 0 (Table 1A).

**Table 1:** Two-point LOD scores of chromosome 11q markers for families A) PKCC001 and B) PKCC113. The asterisk indicates marker included in the genome-wide scan.

Family ID	Marker	cM	Mb	0.00	0.01	0.05	0.10	0.20	0.30	0.40	*Z*_max_	θ_max_
**A**												
PKCC001	D11S4175*	91.47	90.25	∞	-2.13	-0.14	0.54	0.85	0.69	0.34	0.85	0.20
PKCC001	c.34C>T		111.78	5.53	5.05	4.54	3.45	2.27	1.04	5.44	5.53	0.00
PKCC001	D11S4078	105.74	112.25	4.29	4.21	3.83	3.35	2.33	1.30	0.39	4.29	0.00
PKCC001	D11S925*	118.47	120.82	∞	0.87	1.92	2.08	1.75	1.13	0.47	2.08	0.10
**B**												
PKCC113	D11S4175*	91.47	90.25	0.91	0.88	0.76	0.62	0.34	0.13	0.02	0.91	0.00
PKCC113	c.31C>T		111.78	1.63	1.59	1.41	1.19	0.75	0.37	0.12	1.63	0.00
PKCC113	D11S4078	105.74	112.25	1.21	1.17	1.00	0.80	0.41	0.13	0.02	1.21	0.00
PKCC113	D11S925*	118.47	120.82	1.18	1.15	1.00	0.83	0.51	0.26	0.10	1.18	0.00

**Fig 3:**
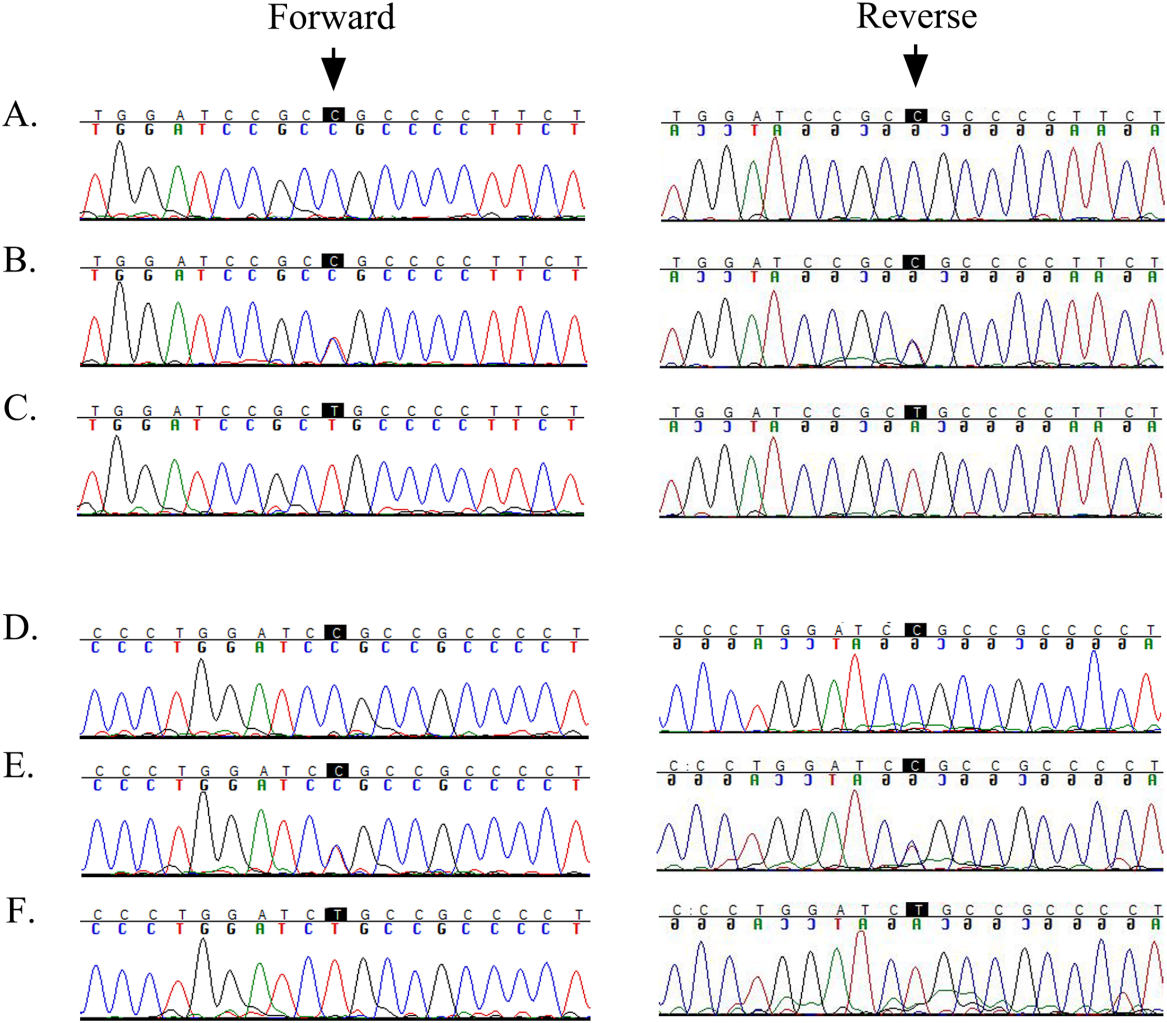
Sequence chromatograms of causal mutations identified in PKCC001 and PKCC113. Sequence chromatograms of A) Unaffected individual 15 homozygous for wild-type allele; B) unaffected individual 17 heterozygous and C) affected individual 19, homozygous for c.34C>T (p.R12C). Sequence chromatograms of D) unaffected individual 6 homozygous for wild-type allele, E) unaffected individual 8 heterozygous and F) affected individual 10 homozygous for c.31C>T (p.R11C). The arrows point to c.31C and c.34C of *CRYAB* mutated in PKCC113 and PKCC001, respectively. It is worth to note that mutations identified in PKCC001 and PKCC113 are adjacent amino acids i.e. Arg11, and Arg12.

This region harbors *CRYAB*, a gene previously associated with cardiomyopathy and congenital cataracts. Bi-directional sequencing of *CRYAB* identified a missense variation: c.34C>T; p.R12C that segregated with the disease phenotype in PKCC001 (Figs 1A and 3A–3C). The exome variant server analysis identified the variant in heterozygous form (AA = 0/AG = 1/GG = 4295) in one individual representing a minor allele frequency (MAF) of 0.0116 in European American population. Likewise, dbSNP analysis revealed MAF score of 0.0010 for rs375933774 (c.34C>T) based on 1000 Genomes database. This variation was not found in 384 control chromosomes of Pakistani and 48 control chromosomes of Saudi decent. A two-point LOD score of 5.53 at θ = 0 was obtained for the causal variant (Table 1A).

To estimate the total genetic load of *CRYAB* in our cohort of familial cases, we interrogated our cohort of >200 familial cases of congenital cataracts by genotyping closely-spaced STR markers followed by sequencing all coding exons of *CRYAB*. We identified one additional family, PKCC113 linked to the chromosome 11q23 (Fig 1B) with positive two-point LOD scores (Table 1B). Bi-directional sequencing of *CRYAB* identified a novel missense mutation: c.31C>T (p.R11C) that segregated with the disease phenotype within the family (Fig 3D–3F). Likewise, this variation was not found in 384 and 48 control chromosomes of Pakistani and Saudi decent, respectively.

**Fig 4:**
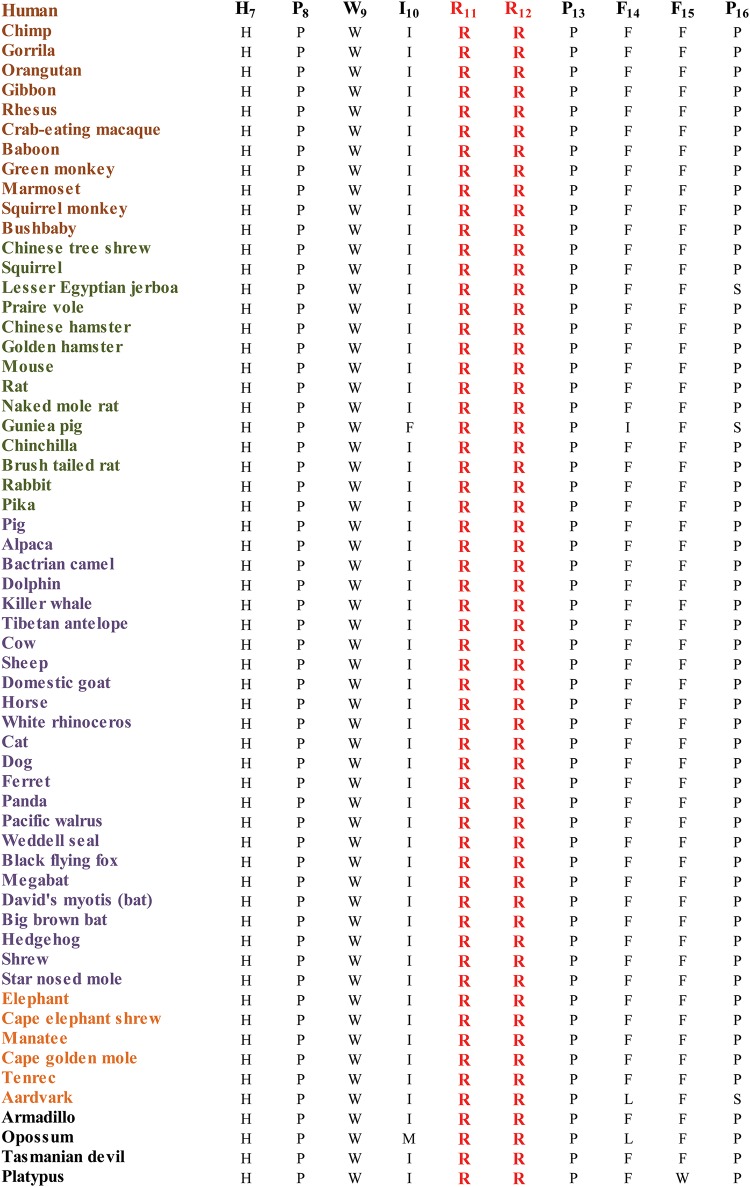
Sequence alignment of *CRYAB* in mammalian orthologues illustrating the conservation of Arginine at positions 11 and 12. Primates, Euarchontoglires, Laurasiatheria, and Afrotheria are colored brown, green, purple and orange, respectively.

We used SIFT and PolyPhen2 algorithms to evaluate the possible impact of p.R11C and p.R12C mutations on CRYAB. SIFT predictions suggested that both the R11C and R12C substitutions will not be tolerated by the native three-dimensional structure of CRYAB. The effect protein function score for R11C and R12C were 0.00 and 0.00, respectively (amino acids with probabilities < .05 are predicted to be deleterious). Likewise, Polyphen2 suggested that both the R11C and R12C substitutions are probably damaging to the CRYAB structure with a score of 1.00, and 1.00, respectively. We found that both arginines at position 11, and 12 are not only conserved in CRY*AB* mammalian orthologues (Fig 4) but also conserved in CRYAB vertebrate orthologues according to the UCSC genome browser (data not shown).

**Fig 5:**
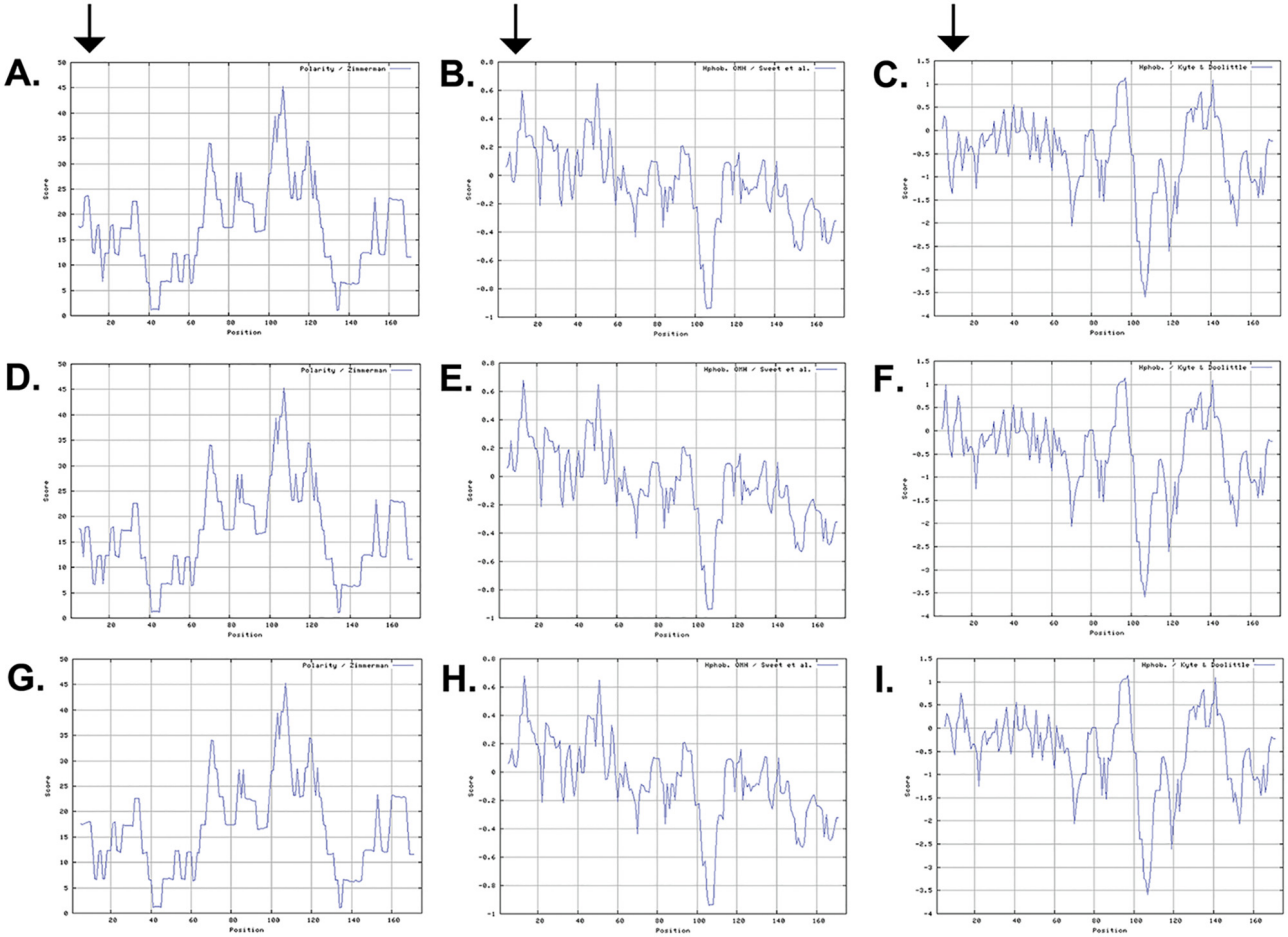
Investigating the physical characteristics of wild-type and mutant *CRYAB* proteins. The polarity (A, D, G), the optimized matching hydrophobicity (B, E, H), and hydropathicity (C, F, I) plots of the wild-type and mutant CRYAB proteins. Both mutant proteins (R11C and R12C) revealed low polarity (compare A, with D and G), a higher hydrophobicity (compare B, with E and H), and higher hydropathicity (compare C, with F and I), respectively. The x-axis represents the position of amino acids. The y-axis represents the Polarity, hydrophobicity and Hydropathicity values in a default window size of 9. The arrows point to the difference in their respective polarities (1^st^ arrow from the left), hydrophobicity (2^nd^ arrow from the left) and hydropathicities (3^rd^ arrow from the left).

Subsequently, we examined the impact of these mutations on the physical characteristics of CRYAB. The ProtScale software predicted lower polarity, higher hydrophilicity and hydrophobicity of the mutant CRYAB compared to the wild type residues in protein secondary structure (Fig 5). In parallel, we used mCSM, a structure-based algorithm to validate the damaging nature of both missense variants identified in CRYAB. The analysis predicted the destabilizing nature of both variants (R11C and R12C) that would result in the disruption of the secondary structure of the protein (Table 2). Finally, we estimated the isoelectric point (pI) and computed molecular weight of the mutant CRYAB proteins. We found that both mutant CRYAB proteins had a lower pI (pI: 6.5) compared to the wild type CRYAB (pI: 6.76).

**Table 2:** The predictive changes of *CRYAB* missense variants on the protein stability. RSA: Residue Relative Solvent Accessibility, ΔΔG**:** Reduction in free energy.

PDB File	Chain	Wild-type Residue	Residue Position	Mutant Residue	RSA	Predicative ΔΔG (Kcal/mol)	Outcome
2YGD.pdb	A	R	11	C	48.9	-1.576	Destabilizing
2YGD.pdb	A	R	12	C	42.5	-1.448	Destabilizing

**Fig 6:**
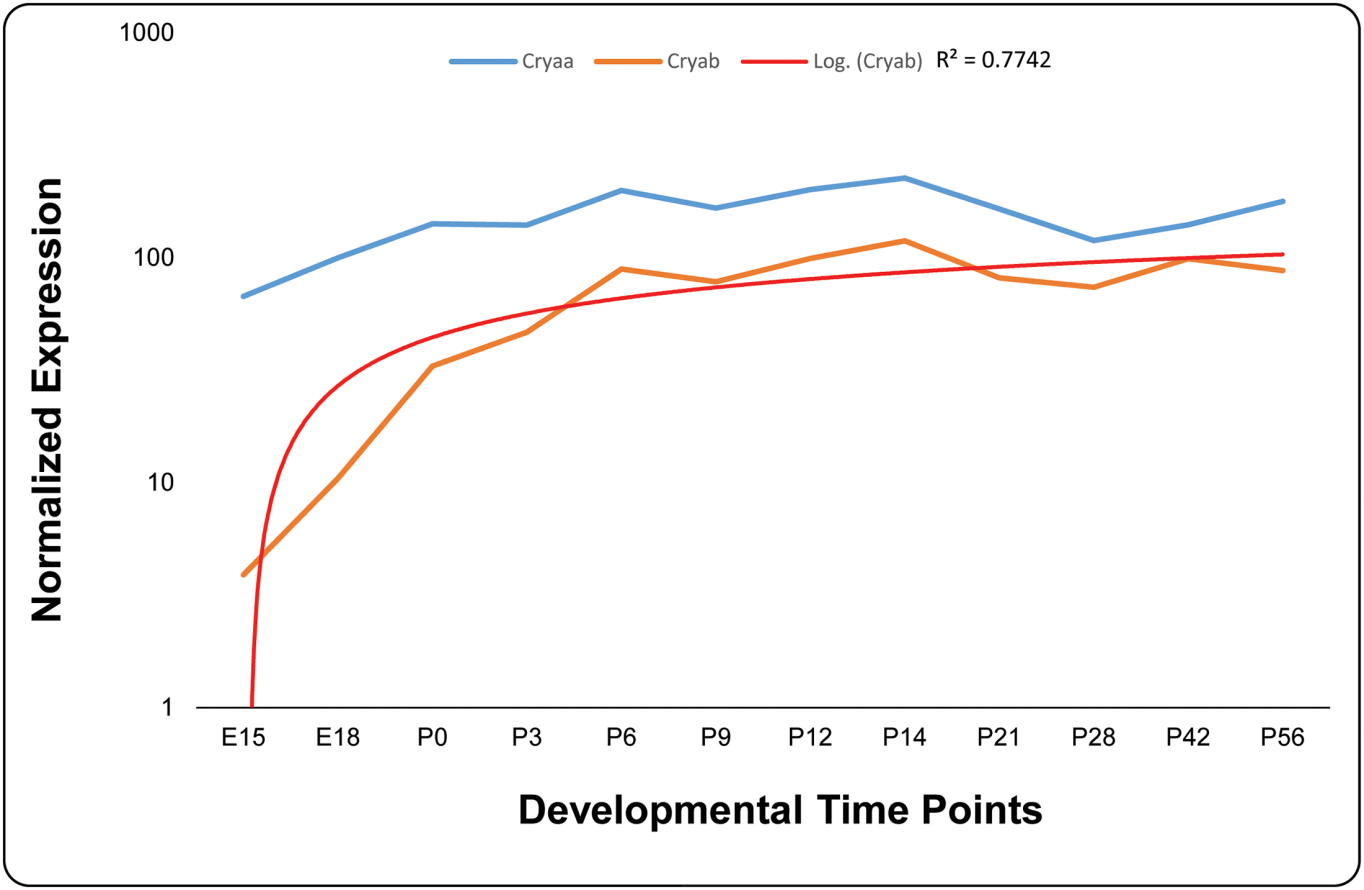
Expression profile of alpha-crystallin in developing mouse lens. The expression of *Cryaa* (blue) and *Cryab* (orange) at different developmental time points was normalized to *Gapdh*. A logarithmic trend line (red) fits the *Cryab* expression with an R^2^ value of 0.7742. The x-axis and y-axis represent developmental time points and normalized expression of each mRNA, respectively.

Dubin and colleagues, previously reported the expression of *CRYAB* in multiple tissues including the ocular lens [22]. We investigated the expression of both *Cryaa* and *Cryab* in embryonic and postnatal murine lens. As shown in Fig 6, we observed expression of both α-Crystallins in mouse lens as early as embryonic day 15 (E15); nonetheless, the level of *Cryaa* expression was an order of magnitude higher compared with expression of *Cryab*. In sharp contrast to *Cryaa* of which the expression levels remain nearly steady over the 12 developmental stages investigated here, the expression level of *Cryab* mimics a logarithmic pattern in early stages of increasing significantly up until postnatal day 6 (P6) and from there onwards the expression level remains steady over the remaining time course until two months of age (Fig 6).

## Discussion

Here, we report two novel mutations in *CRYAB* associated with autosomal recessive congenital cataracts identified in consanguineous Pakistani families. Initially, a genome-wide linkage scan localized the critical interval to chromosome 11q23 in PKCC001 while sequencing of the coding exons of *CRYAB* identified a novel missense mutation. Subsequently, we identified a second family, PKCC113 harboring a second novel missense mutation in *CRYAB*. While both mutations segregated with the disease phenotype in their respective families, none of these novel variants were present in normal control chromosomes.

Vicart and colleagues reported a missense mutation (p.R120G) in the *CRYAB* associated with a desmin-related myopathy with affected individuals exhibiting signs of hypertrophic cardiomyopathy and discrete lens opacities [26]. Subsequently, multiple heterozygous mutations in different ethnic populations were reported with cardiomyopathy and/or cataractogenesis (Table 3) [26–41]. Among these, Safieh and colleagues reported a missense mutation c.166C>T (p.R56W) associated with autosomal recessive cataracts in a consanguineous family of Saudi decent with no clinically significant myopathy [36]. However, one of the older affected individuals presented symptoms of retinal dystrophy and in the later publication the authors showed evidence of clinical rod-cone degeneration only in the adults homozygous for the R56W allele who were aphakic since childhood [37]. We did not observe any retinal abnormalities in individuals homozygous for R11C and R12C alleles during a follow-up visit; nonetheless, we cannot rule out the possibility that affected individuals of PKCC001 and/or PKCC113 may develop retinal phenotype in the later years of their lives.

**Table 3:** A list of causal mutations reported in *CRYAB* associated with congenital cataracts and cardiomyopathy. adCC: autosomal dominant congenital cataracts; arCC: autosomal recessive congenital cataracts; DCM: dilated cardiomyopathy; MM: myofibrillar myopathy.

No.	Nucleotide change	Amino Acid Change	Coding Exon	Associated Pathology	Reference
1	c.32G>A	Arg11His	1	adCC	[35]
2	c.31C>T	Arg11Cys	1	arCC	Current study
3	c.34C>T	Arg12Cys	1	arCC	Current study
4	c.59C>G	Pro20Arg	1	adCC	[26]
5	c.58C>T	Pro20Ser	1	adCC	[31]
6	c.166C>T	Arg56Trp	1	arCC	[36,37]
7	c.205C>T	Arg69Cys	1	adCC	[39]
8	c.325G>C	Asp109His	2	adCC, CM	[40]
9	C358A>G	Arg120Gly	2	adCC	[40]
10	c.418G>A	Asp140Asn	3	adCC	[32]
11	c.450delC	Lys150Asnfs34*	3	adCC	[27]
12	c.451C>T	Gln151*	3	MM	[28]
13	c.460G>A	Gly154Ser	3	DCM	[33]
14	c.464_465delCT	Pro155Argfs9*	3	MM	[28]
15	c.470G>A	Arg157His	3	DCM	[30]
16	c.514G>A	Ala171Thr	3	adCC	[34]
17	c.527A>G	Ter176Trpfs19*	3	adCC, DCM	[41]

Ghosh and colleagues, recently identified seven interactive sequences for CRYAB chaperone activity through protein pin arrays, which included two sequences in the N-terminal domain, four in the crystallin core domain and one in the C-terminal domain [29]. Interestingly, the first interactive sequence in the N-terminal domain comprising of amino acids 9–20 (WIRRPFFPFHSP) includes Arg11 and Arg12, the two amino acids mutated in PKCC113 and PKCC001, respectively. Taken together, it is conceivable that these two causal mutations (p.R11C and p.R12C), which substitute a positively charged amino acid for a non-polar amino acid will most likely distort the electrostatic balance of CRYAB. Given the fact that heterozygous carriers of these mutations are phenotypically normal, it is safe to assume that both variations render the protein functionless with no gain-of-function or dominant negative effect.

Recently, Chen and colleagues reported a missense mutation involving Arg11, an arginine to histidine substitution (R11H) responsible for autosomal dominant congenital nuclear cataracts. The mutation responsible for a dominant phenotype results from a positively charged arginine substituting for a positively charged histidine while the recessive phenotype results from polar cysteine substitution. Nevertheless, the biophysical characteristics of the p.R11C mutant CRYAB (Fig 5 and Table 2) are not much different compared with p.R11H mutant protein [35]. The precise mechanism of different inheritance patterns resulting from different substitutions for a particular amino acid remains elusive and warrant additional biochemical analyses to decipher the mechanism responsible for different inheritance patterns.

In conclusion, we report two mutations in *CRYAB* associated with autosomal recessive congenital cataracts. Identification of causal variants associated with cataractogenesis will help us better understand the biology of the ocular lens including mechanistic details of the maintenance of lens transparency.
