# Correction: Missense Mutations in *CRYAB* Are Liable for Recessive Congenital Cataracts

**DOI:** 10.1371/journal.pone.0171403

**Published:** 2017-01-30

**Authors:** Xiaodong Jiao, Shahid Y. Khan, Bushra Irum, Arif O. Khan, Qiwei Wang, Firoz Kabir, Asma A. Khan, Tayyab Husnain, Javed Akram, Sheikh Riazuddin, J. Fielding Hejtmancik, S. Amer Riazuddin

The first author’s name is spelled incorrectly. The correct name is: Xiaodong Jiao. The correct citation is: Jiao X, Khan SY, Irum B, Khan AO, Wang Q, Kabir F, et al. (2015) Missense Mutations in *CRYAB* Are Liable for Recessive Congenital Cataracts. PLoS ONE 10(9): e0137973. doi:10.1371/journal.pone.0137973

## References

[1] Jiaox X, Khan SY, Irum B, Khan AO, Wang Q, Kabir F, et al (2015) Missense Mutations in *CRYAB* Are Liable for Recessive Congenital Cataracts. PLoS ONE 10(9): e0137973 doi: 10.1371/journal.pone.0137973 26402864 10.1371/journal.pone.0137973 PMC4581838

